# Team approach to polypharmacy evaluation and reduction: study protocol for a randomized controlled trial

**DOI:** 10.1186/s13063-021-05685-9

**Published:** 2021-10-26

**Authors:** Dee Mangin, Larkin Lamarche, Gina Agarwal, Hoan Linh Banh, Naomi Dore Brown, Alan Cassels, Kiska Colwill, Lisa Dolovich, Barbara Farrell, Scott Garrison, James Gillett, Lauren E. Griffith, Anne Holbrook, Jane Jurcic-Vrataric, James McCormack, Daria O’Reilly, Parminder Raina, Julie Richardson, Cathy Risdon, Mat Savelli, Diana Sherifali, Henry Siu, Jean-Éric Tarride, Johanna Trimble, Abbas Ali, Karla Freeman, Jessica Langevin, Jenna Parascandalo, Jeffrey A. Templeton, Steven Dragos, Sayem Borhan, Lehana Thabane

**Affiliations:** 1 Department of Family Medicine, McMaster University, 100 Main Street West., 5th floor, Hamilton, Ontario L8P 1H6 Canada; 2 University of Alberta, 6-60 University Terrace, Edmonton, Alberta Canada; 3 University of Victoria, 3800 Finnerty Road, Victoria, BC Canada; 4 University of Toronto, 144 College Street, Toronto, Ontario Canada; 5 Bruyère Research Institute, 43 Bruyère Street, Ottawa, Ontario Canada; 6 University of British Columbia, 2405 Wesbrook Mall, Vancouver, BC Canada

**Keywords:** Polypharmacy, Overmedication, Multimorbidity, Deprescribing, Older adults, Medication reduction, Health outcomes, Operationalized clinical model, Physician pharmacist collaboration

## Abstract

**Background:**

Polypharmacy in older adults can be associated with negative outcomes including falls, impaired cognition, reduced quality of life, and general and functional decline. It is not clear to what extent these are reversible if the number of medications is reduced. Primary care does not have a systematic approach for reducing inappropriate polypharmacy, and there are few, if any, approaches that account for the patient’s priorities and preferences. The primary objective of this study is to test the effect of TAPER (Team Approach to Polypharmacy Evaluation and Reduction), a structured operationalized clinical pathway focused on reducing inappropriate polypharmacy. TAPER integrates evidence tools for identifying potentially inappropriate medications, tapering, and monitoring guidance and explicit elicitation of patient priorities and preferences. We aim to determine the effect of TAPER on the number of medications (primary outcome) and health-related outcomes associated with polypharmacy in older adults.

**Methods:**

We designed a multi-center randomized controlled trial, with the lead implementation site in Hamilton, Ontario. Older adults aged 70 years or older who are on five or more medications will be eligible to participate. A total of 360 participants will be recruited. Participants will be assigned to either the control or intervention arm. The intervention involves a comprehensive multidisciplinary medication review by pharmacists and physicians in partnership with patients. This review will be focused on reducing medication burden, with the assumption that this will reduce the risks and harms of polypharmacy. The control group is a wait list, and control patients will be given appointments for the TAPER intervention at a date after the final outcome assessment. All patients will be followed up and outcomes measured in both groups at baseline and 6 months.

**Discussion:**

Our trial is unique in its design in that it aims to introduce an operationalized structured clinical pathway aimed to reduce polypharmacy in a primary care setting while at the same time recording patient’s goals and priorities for treatment.

**Trial registration:**

Clinical Trials.gov NCT02942927. First registered on October 24, 2016.

**Supplementary Information:**

The online version contains supplementary material available at 10.1186/s13063-021-05685-9.

## Background

There is increasing concern about the harms of inappropriate medication use. Data suggest that adverse drug reactions may account for more morbidity and mortality annually than the potential benefit for the many chronic diseases the medications are designed to treat [1–5]. The death rates from adverse drug effects are higher than those from many common cancers [4, 6]. There are particular concerns around polypharmacy in older adults. While there are varying definitions of polypharmacy, the most common definition is use of 5 or more long-term medications [7]. Polypharmacy is associated with negative health outcomes in older adults including falls, impaired cognition, and poorer nutrition [8–11]. Other potential negative associations include drug-drug interactions, drug-disease interactions, reduced medication adherence and difficulties in managing complicated medication regimes which exceed the patients’ ability to cope [5, 10, 12–14].

The evidence for the effect of interventions to reduce polypharmacy on health outcomes shows inconsistent results, and difficulties in implementation; several reviews recommended further randomized controlled trials (RCTs) evaluating multidisciplinary interventions and clinical outcomes across different settings [10, 15–20]. Our feasibility study in 2010, a prospective cohort study of multiple medication discontinuation in community-dwelling adults, showed medication reduction and positive effects on health outcomes with no serious adverse effects [21]. Furthermore, a feasibility study in long-term care, targeting deprescribing of anticholinergic and sedative medicines, showed that medication reduction resulted in reductions in psychotropic drug side effects, falls, and depression and frailty scores [22]. Evidence shows that use of explicit lists of potentially inappropriate medicines (PIMs), such as the Screening Tool of Older Person’s Prescriptions (STOPP) and Beers list criteria can improve appropriateness of prescribing in older adults [23–25]. As well, potential benefits of using explicit PIMs list for predicting adverse drug events have been found [26–30]. However, these studies provide insufficient evidence of the reversibility of the negative health effects associated with polypharmacy.

A number of proposed approaches to polypharmacy, broadly divisible into implicit and explicit approaches, have been reviewed in detail in a previous publication [10] and so they will not be discussed here. The tools available are largely used as decision aids to guide clinicians on which medicines are likely to be inappropriate in older patients or to cause adverse events, based on observational data and consensus [31]. More recently, electronic deprescribing aids that aggregate multiple sources of evidence have been developed. These and other screening tools have been developed from observational studies. These tools are typically explicit lists of individual PIMs, and indices of cumulative side effect burden which help to identify some problematic medications that are associated with hospitalizations due to adverse drug events, increased length of hospital stay, and increased risk and rate of falls [26–28, 32, 33]. Deprescribing guidelines have also been developed for some individual classes of medications, providing evidence-based decision algorithms as a pathway to reducing or stopping these medications in these classes under specific circumstances [34–38].

Explicit screening tools, while useful, are not sufficient as a complete approach to reducing harms of polypharmacy as they include by necessity only the most common, adverse drug events, or focus on selected adverse effects [39]. Further, such tools do not seek information directly from patients about side effects nor consider patient-focused prioritization [40]. Therefore, we carried out a systematic review of operationalized models recording patient preferences for medication use in the context of multimorbidity or polypharmacy [40]. We found no such models, though we did find a part of one tool that was valuable, as it assessed relative prioritization of symptom treatment versus treatments to prevent future illness, and has been validated in a similar population [41].

### Team Approach to Polypharmacy Evaluation and Reduction (TAPER)

Primary care is strongly associated with improved health outcomes and is highlighted as an ideal setting for addressing polypharmacy: its generalist, comprehensive, person-focused approach to care, which offers integrated and longitudinal continuity [1]. However, there are no systematic approaches for family practice that are routinely embedded in clinical care for reducing inappropriate polypharmacy, and no interventions we are aware of that take a conceptual approach based on these core principles of primary care, and the core functions that support the effect of primary care on improved health outcomes (first contact care, comprehensiveness, continuity, person focus). A feasibility RCT of a Team Approach to Polypharmacy Evaluation and Reduction (TAPER) in a primary care setting in Canada similarly showed medication reduction and that the direction of most of the outcome measures supported the effectiveness of TAPER compared to usual care. This preliminary exploration supporting positive trends in effect across a variety of outcome measures [42] provide the support needed to proceed with this larger RCT powered to test the effects of TAPER, and to determine whether it can reduce medications numbers and affect health outcomes.

In this study, we propose to test TAPER, an intervention designed to be suitable for routine use in primary care. TAPER is seated within a theoretical framework of prevention (Quaternary Prevention, or “P4”), which includes actions designed to prevent the harms of unnecessary medication use, overtreatment, and over-dosage [43]. It is designed to address common barriers to reducing polypharmacy identified in the literature, such as lack of knowledge regarding deprescribing, patient fears of not being able to restart medications, physician and patient unwillingness to initiate conversations about medication reduction, physician uncertainty about how to conduct goals of care conversations, lack of evidence support or a clear pathway for approaching polypharmacy, and a focus on providing care congruent with multimorbidity rather than disease-specific clinical guidelines [44–47]. TAPER conforms to standards for high quality acronyms (score 14.5) [48]. The model development and its theoretical basis will be described in a separate publication [45]; some further details are provided in the “Methods” section.

In brief, TAPER is a structured operationalized clinical pathway for comprehensive multidisciplinary collaborative medication review by the team including pharmacists and physicians in partnership with patients. The clinical pathway is structured around the core functions and principles of the primary care model and focuses on reducing medication burden, and through this, potentially the harms of unnecessary polypharmacy. TAPER is designed to explicitly overcome known barriers to polypharmacy reduction which includes patient fears of not being able to restart medications, physician/pharmacist and patient unwillingness to initiate conversation, physician/pharmacist uncertainty about how to conduct goals of care conversations, lack of evidence support or a clear pathway, lack of confidence in tapering and deprescribing, lack of teamwork, and lack of a mode for communication between pharmacist and physician [45]. The mapping of these is described explicitly and in more detail in a paper describing the theoretical model for TAPER [40]. TAPER sequentially links consultations with first a pharmacist and then a family physician and frames the medication reduction pathway as a structured “pause and monitor” drug holiday period. Evidence support tools are integrated within an underpinning secure digital platform (TaperMD) [49] to automatically flag potentially inappropriate medications and cumulative medication burdens. Patient priorities are explicitly sought across several domains. TaperMD serves to integrate the consultation elements, patient priorities and preferences, and evidence supports across providers in a shared electronic record. This platform is designed for incorporation into clinicians' existing electronic medical record systems.

## Methods

### Aims

We will use an RCT to assess the effects of implementing the TAPER model in addressing polypharmacy and on a range of health-related outcomes and health service use, in adults 70 years of age or older in a primary care setting. A secondary aim is to qualitatively explore the experience of reducing medications using this approach for patients, family physicians, and pharmacists.

### Research design

We will conduct a 1:1 single blind RCT. Participants in the control group will be offered the intervention after 6-month research outcomes are assessed (wait list control).

### Ethics, registration, and trial guideline adherence

At the time of submission, ethics approval was granted by the Hamilton Integrated Research Ethics Board (16-November-2016; project file #2226), the University of Alberta’s Health Research Ethics Board (21-August-2019; project file # Pro00090823), and the Vancouver Island Health Authority Clinical Research Ethics Board (06-January-2020; project file #C2019-075). The trial is registered with ClinicalTrials.gov (NCT02942927), which includes all items from the World Health Organization Trial Registration Data Set. We used the SPIRIT guidelines to inform reporting of our trial protocol (Additional File 1) [50]. We also used the TIDieR checklist for intervention description and replication (Additional File 2) [51]. Important protocol modifications will be tracked and communicated through an ethics amendment and ClinicalTrials.gov. Consent form and other related documentation given to participants and authorized surrogates available upon request.

### Trial setting

The trial will take place in three Canadian primary care locations. The lead implementation site is the McMaster University Practice Based Research Network (McMaster University Sentinel and Information Collaboration) which serves a population of 50,000 patients [1, 52]. Secondary sites will be in Alberta (Kaye Edmonton Clinic, over 7000 patients rostered) and British Colombia (multiple clinics in the Nanaimo Health Region).

### Participants

The source population includes patients living in Canada who have a regular family physician. All patients rostered with participating family physicians are eligible for participation provided they fit inclusion and exclusion criteria.

Patients meeting the following criteria will be eligible for the trial:

- Aged 70 years of age or older
- Taking 5 or more long-term prescribed medications (excluding topical products) [7]
- Rostered to a family physician participating in the trial
- Willing to try discontinuation

Patient will be excluded based on the following exclusion criteria:

- Unable to understand English or do not have cognitive skills to understand and respond to rating scales
- Terminal illness or other circumstance precluding the 6-month study period follow-up
- Had a comprehensive medication review within the past 12 months

### Participant recruitment

Patients will be identified through automated screening by participating family physicians’ electronic medical record (EMR) to find patients 70 and over and taking 5 or more long-term medications. Potentially eligible patients will be sent a letter from their family physician outlining the study. If patients are interested in participating, they will be asked to either reply in a postage paid envelope to the study team or call the study phone number. They will then be contacted by a member of the study team who will go through a formal information, consent process, and eligibility screen. Patients who consent will then have the study appointments made according to their allocation to intervention or control group.

### Allocation and randomization

Patients will be randomly allocated 1:1 to either the intervention or control group using an internet accessible computerized system [53]. Randomization will be blocked and stratified by site. The randomization sequence will be generated centrally using a computerized system and will be maintained external to the research team. The Biostatistics Unit at St. Joseph’s Healthcare Hamilton will be responsible for generating the sequence.

### Blinding

Patients will not be blinded to the allocation as this would require preparation of identical placebos for all medications and tapering regimes and is not practically or economically feasible. This is a trial of effectiveness of reducing the number of medications, rather than a trial of the chemical effect of a drug where an identical placebo control is appropriate. The use of placebos in this planned trial would also negate the ability to look at the effects on treatment burden and medication self-efficacy that may be associated with simplification of medication regimes.

Outcome assessment will be blinded with “quarantine” methods developed in the feasibility RCT [42] to maintain blinding of the patient outcome assessor. The effectiveness of this blinding will be assessed at study end by asking the outcome assessor to guess which arm the patient is in, after outcome measures have been evaluated. Pharmacists and family physicians will be masked to allocation to some extent, in that they will not be aware of whether the appointment they are carrying out is for an intervention participant or 6-month waitlist control participant. Pharmacists and family physicians will only be able to access TaperMD and its tools for enrolled patients immediately prior to their appointments with individual patients. The data analysts will be blinded to group allocation; however, they will know the number of groups there are.

### Intervention

We developed TAPER as a multifaceted intervention that integrates available evidence tools, a team approach and technology within a clinical pathway where decision making is led by consideration of patient priorities. Barriers to addressing polypharmacy identified in the literature, and in our formative work for this trial, were considered in its development [44, 46, 54–57]. Participatory-based methods were used with consumers, pharmacists and family physicians to inform the approach and development of the tool so the intervention is as “fit for purpose” and as feasible as possible. We also carried out a feasibility RCT as phase 1 of this project to inform and revise the model (reported separately) [42]. To foster fidelity, participating pharmacists and family physicians will receive an in-person training session for TaperMD and the entire intervention. A demonstration video and manual will also be provided.

TAPER will be operationalized as a structured clinical pathway in an electronic web-based platform, called TaperMD (see Additional File 3 for details), which records and integrates the following:

1. *Patient priorities for treatment*: Explicit recording of patient preferences and priorities occurs first in TaperMD. Question domains cover symptom priorities, functional priorities, medication experience and preferences including ranking of the importance of symptomatic and preventive treatments, financial challenges around medications, and perceived medication burden [40, 41, 56].
2. *Patient characteristics relevant to medications* including falls history, blood pressure, and renal function (may be reported by patient, pharmacist, or physician).
3. *Patient medications* (prescribed and over the counter), indication, including explicit questions for each medication from the pharmacist on any self-reported perceived side effects.
4. *Evidence-based screen for potentially inappropriate medications*: The medications recorded in #3 (Patient medications) will be integrated into an automatic electronic screen for flagging potentially inappropriate medications or cumulative medication burdens in older adults. These flags will detail the underpinning evidence or recommendation and provide access to any available evidence tools at the “point of care.” This evidence is supportive rather than prescriptive, as the model is designed to be led by the patient’s priorities and preferences for treatment, as well as their experience of medication benefits and side effects. These tools and lists highlight potentially inappropriate medicines (and reasons) in older adults in a simultaneous multi-drug view consistent with a multimorbidity approach. Specific medication dimensions flagged will include standard interaction checking, potentially inappropriate medicines in older adults drawn from assessment of a wide range of jurisdiction specific lists [10], drugs contributing to anticholinergic burden score, QT prolonging drug burden, hypotensive drug burden, serontonergic drug burden, and deprescribing guidelines and algorithms where these are available. These are described in detail in Additional File 3.
5. *Recommendations* for drug classes around tapering approaches, potential medication withdrawal effects, and suggested monitoring parameters and time frames will be provided to support tapering and/or deprescribing.
6. One of the barriers to collaboration between clinicians is often the absence of communicating electronic medical records between providers (in particular pharmacists and prescribers). TaperMD serves as a *shared record for all clinicians* in the pathway to access and review. It is designed to be integrated with the electronic medical record of both pharmacists and family physicians, other relevant clinicians through an application programming interface (API) to an EMR, or printed for integration with paper records of clinicians and patients. It has been designed to also allow for future patient access.

### Operational steps

#### STEP 1: Collection of patient information

**Fig. 1:**
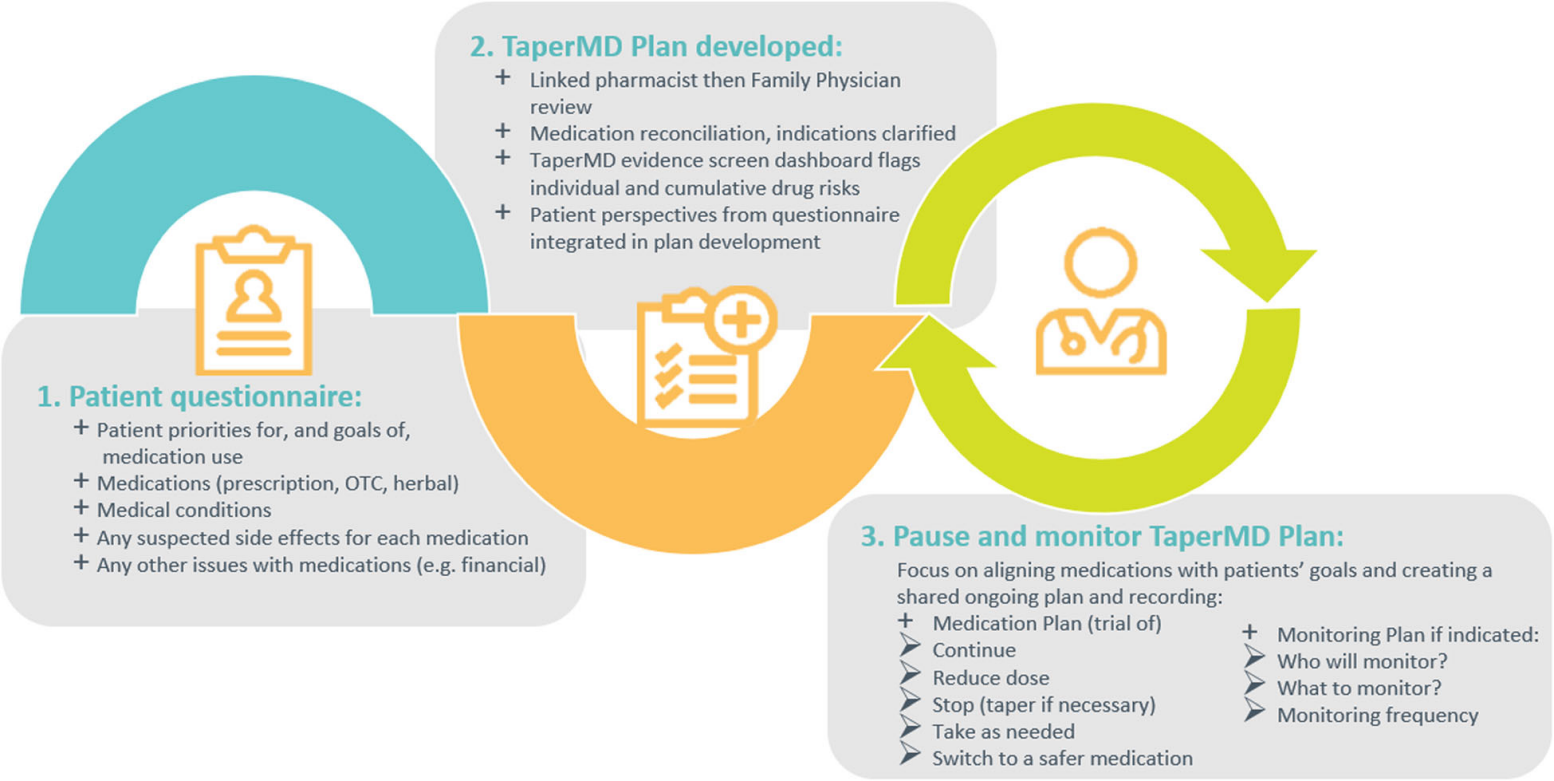
Operational steps of TaperMD

Patient priorities and any available data on patient characteristics relevant to medications will be entered into TaperMD. For the study, this will be done by the research assistant along with the collection of baseline outcome data; however, the tool is designed for future patient/family access to complete this step (Fig. 1).

#### STEP 2: Pharmacist consultation

The patient will meet first with the pharmacist to review the list of medications for reconciliation with what the patient reports they are taking, including accuracy of dose and frequency and indications. Specific side effect inquiry is recorded for each medication as well as any further relevant patient characteristics. A medication review will be done, focused on medications suitable for stopping or dose reduction, supported by the TaperMD screen and evidence resources for tapering, the reported medication side effects from the patient, and review of the patient’s medication experience and priorities and preferences for treatment.

The pharmacist will make initial recommendations focused on medications suitable for discontinuation or dose reduction and record these in TaperMD, which will then be visible to the family physician. The summary of this initial plan will also be integrated into the pharmacist’s electronic record.

#### STEP 3: Family physician consultation

Within a maximum of 2 weeks later, the patient will have an appointment with their family physician to discuss the pharmacist recommendations and identify medications that may be suitable for a “pause and monitor” phase of discontinuation or dose reduction. The family physician can access and view the TaperMD record prior to the consultation (patient priorities, patient characteristics relevant to medications, evidence screen, recorded potential medication side effects, and pharmacist recommendations for each medication). The family physician will verbally review the patient’s priorities and preferences, and incorporate their knowledge of the patient over time, their context, and their medical conditions in this review of the pharmacist’s recommendations and discussion with the patient. Patient goals and priorities can be adjusted at this point, based on these discussions.

Every medication is reviewed and a final recommendation is recorded to either continue, stop, reduce the dose, or switch to a safer medication. The discontinuation and follow-up monitoring plan is finalized including timing of changes (not all may occur immediately if a number are being stopped), tapering and any agreed criteria for restarting. TaperMD generates a summary of the plan (a TAPER plan), including the tapering, monitoring, and follow-up plan. This will be pulled into the usual clinical record (e.g., EMR) as the visit record and can be printed for the patient and their family.

Due to the COVID pandemic, patients were given the option to have in person or virtual/video consultations.

### Monitoring

Study participants will be followed for 6 months. Participants will attend monitoring visits, as clinically indicated, with the most appropriate clinician during the “pause and monitor” phase and informed by the tools supporting tapering and monitoring. Planned monitoring visits will be recorded in TaperMD and pulled into the EMR as the visit record. Participants will have no restrictions on other clinical activities.

### Control group

The control group will receive usual clinical care. The only restriction is there will be no other planned medication reviews performed in the primary care setting during this phase. Patients carry information requesting no routine medication deprescribing settings for the 6-month period, except if medically indicated. Control patients will be offered the intervention after their 6-month outcome assessment. This comparator was chosen as it was felt this approach was likely to reduce contamination bias and maximize recruitment.

### Data collection

#### Patient characteristics

Patient characteristics that include age, gender, household income, ethnicity, and education will be collected at baseline. Also, a measure of comorbidity [58], medication risk [59], frailty [60], and beliefs about medicines [61] will be completed. Patient preferences, priorities, and goals are recorded as part of the clinical intervention.

### Outcome measures

**Fig. 2:**
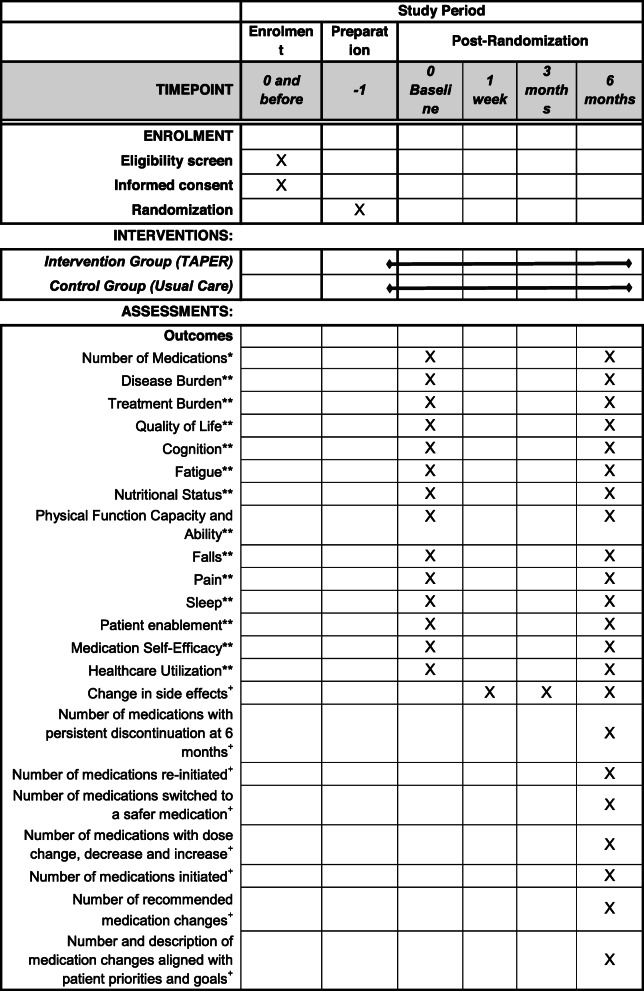
SPIRIT figure with study timeline and data collection time points

Primary and secondary outcomes will be measured at *T*_0_ and *T*_6_ for effectiveness analyses (Research Questions 1 and 2, Fig. 2). See Additional File 4 for a detailed description of the outcome measures and how they relate to the data analysis plan respectively, and Additional File 5 for the detailed schedule of all data collection. Choice of measures was guided by recommendations from an international consensus that identified a set of outcomes specific for multimorbidity patients [62], evidence of their relationship to polypharmacy [8, 9, 12], and piloting of a number of measures in our feasibility RCT (reported elsewhere) [42].

#### Primary outcome measure

The primary outcome includes the number of prescribed medications per patient at baseline and 6 months (see Additional File 4 for definition). Locally acting topical agents without substantial systemic absorption or effects are not included. Acute short-course medications such as antibiotic prescriptions are not included in the counts. This will be ascertained by a pharmacist by performing a medication reconciliation using dispensing information, the electronic medical record, and patient information. Discrepancies will be resolved by discussing with the patient and dispensing pharmacist.

#### Secondary outcome measures

Unless specified, all outcome measures are recorded at baseline and 6 months (see Additional File 4). Constructs included as secondary outcomes include disease burden, treatment burden, quality of life, cognition, fatigue, nutritional status, physical function capacity and ability, falls, pain, sleep, patient enablement, medication self-efficacy, and healthcare utilization.

Other medication-related information will be recorded to identify and describe the processes of medication change during the intervention. Simple descriptive data will be reported on the following process measures:

- Number of medications with persistent discontinuation at 6 months
- Number of medications re-initiated
- Number of medications switched to safer medication,
- Number of medications with dose change (decrease), and dose change (increase)
- Number of medications initiated
- Number of recommended medication changes
- Number and description of medication changes aligned with patient priorities and goals

TaperMD includes a prompt for “completed status” for every medication that includes all these options. Data will be collected through an audit of TaperMD records and progress notes, EMR, and dispensing records. We will describe any observed patterns in groupings of discontinued medications, in particular among those associated with adverse drug reactions causing hospital admissions.

### Adverse events, harms, and beneficial withdrawal effects

Adverse events potentially related to medication withdrawal, potential medication side effects unmasked by discontinuation (i.e., symptom relief), new or worsening side effects, and emergency department visits will be collected by solicited inquiry as well as spontaneous patient or clinician report. The definition from the Common Terminology Criteria for Adverse Events (CTCAE) of Severe/Serious Adverse Event will be used in this study: Any event that requires in-patient hospitalization or prolongation of existing hospitalization, causes congenital malformation, results in persistent or significant disability or incapacity, is life-threatening, or results in death [63]. All adverse events and harms will be summarized and reported with the trial results.

### Check-in data collection

To facilitate accurate recall of adverse events, healthcare utilization, and changes in symptoms or side effects throughout the study duration, telephone check-ins will occur at 1 week, 3 months, and 6 months post-baseline to enquire specifically about these items. Details of any adverse effects identified at these data collection points will be provided in real time to the family physician and pharmacist to address as clinically appropriate. The research ethics board will be notified of any adverse events felt to be study related.

### Patient and provider experience of TAPER

Patients in the intervention arm of the study will be invited to participate in semi-structured interviews to explore the meaning of current medication practices in the context of their everyday lives and their experience of the deprescribing process. Those who respond and consent to this portion will participate in the qualitative study. Attention will be paid to the biopsychosocial experiences of patients in relation to their experiences of the intervention. We will conduct in-depth interviews at the initial stages of the intervention as well as at the end to get a full picture of their experience with the intervention and process of describing.

In addition, this subset of consenting patients will be asked to keep a diary on their chronic disease management practices, and their experience going through the intervention during the study. The aim is to understand both the experience of the intervention and any effects on the complex work of managing multiple chronic diseases. Research on patient compliance suggest that electronic diaries are effective and reliable sources of data [64]. Patients will also be asked about their satisfaction with the intervention process and with their care around medications using Likert type scales, as well as free text responses of the strengths and weaknesses of the process and whether or not they would recommend the intervention to a family member or friend.

Participating pharmacists and family physicians will be asked to record their experiences and feelings about the deprescribing process as field notes during the study. Semi-structured interviews will be conducted at the end of the study at all sites to gain a more in-depth understanding of their experience of the intervention. A single-item question will also be used to measure one’s confidence in their own ability to undertake a process of deprescribing with their patients using a 5-point Likert scale developed for the study, as well as an open-ended question to describe the five best and five worst aspects of the intervention. A validated scale that maps to Normalization Process Theory, the NoMAD survey, is a 23-item survey that will be used to measure implementation processes from the perspectives of those directly involved in implementation of the intervention as the study progresses [65, 66].

### Economic analysis

A 6-month trial-based economic evaluation will be conducted to compare the costs and quality-adjusted life years (QALYs) associated with the intervention and control groups (Research Question 3). Costs will be derived from health care resource utilization data collected in the case report forms and the medical records (e.g., hospitalization, emergency room visits, medication use, home care services, physiotherapy, home care visits). Fixed and variable costs associated with developing and implementing the intervention will be based on trial data. In the base case analyses, Ontario unit costs will be used to cost the healthcare resource utilization and the program costs. To calculate the QALYs, the health utility scores derived from the EQ-5D-5L questionnaire using the Canadian algorithm [67] will be weighted by time spent in health states using an area-under-the-curve approach. Healthcare resource utilization and EQ-5D-5L data will be collected at baseline and at 6 months. Techniques to deal with missing data and censored data will be considered as appropriate [68]. Bootstrap techniques will be used to deal with sampling uncertainty and cost-effectiveness acceptability curves will be used to determine the probability of the intervention to be cost-effective at different willingness-to-pay thresholds ($50,000 or $100,000/QALY gained). The economic evaluation will be conducted according to Canadian [69] and international [68] guidelines and from a Ministry of Health perspective. Because the duration of the study is 6 months, there is no discounting of costs or effects (QALY’s).

### Data management

Table 1 outlines the proposed data analysis methods as they correspond to research questions, hypotheses, and outcome measures. Where appropriate, large font showcards will be used by the researcher to visually present answer options for surveys. Most survey responses will be recorded by the researcher in Research Electronic Data Capture (REDCap) [53, 70], an electronic data capture tool hosted by The Research Institute of St. Joe’s Hamilton, which allows for secure online data entry from multiple sites into a central data repository. Some data that is also relevant to the clinical assessments is recorded in TaperMD and later imported into REDCap. These processes were trialed in our feasibility study [42]. The database has automatic checks and warnings for missing data and will have regular checks for missing data and outliers among data for all sites. Clinical and patient characteristic data from TaperMD will also be imported into REDCap. These processes were tested in our feasibility study [42]. No patient identifiers will be entered into either TaperMD or REDCap, both of which are compliant with both federal and provincial Canadian privacy laws. Patient identifiers will be kept strictly confidential; electronic files containing identifiers will be encrypted and password protected and held in a secure network. Paper documents containing patient identifiers will be held in a locked filing cabinet in a locked room accessible only to members of the study team. Electronic and paper documents will be kept for 10 years.

**Table 1:** Data analysis plan

Research question	Hypothesis	Outcome measure	Timing	Method of analysis
What is the effect of a structured medication discontinuation clinical pathway designed to reduce polypharmacy on mean number of medications and patterns of discontinuation compared to usual practice?	Reduction in mean number of medications	Mean number of medications (primary outcome)	*T*_0_, *T*_6_	Linear regression
		Proportion of participants with successful reduction in medication number or dose	*T*_6_	Logistic regression
		Composite variable of mean number of medication discontinuations and/or dose reductions	*T*_6_	Linear regression
What is the effect of a structured medication discontinuation clinical pathway designed to reduce polypharmacy on patient quality of life, cognition, mobility-related fatigue, nutritional status, physical function capacity, pain, sleep, patient enablement, medication self-efficacy, medication confusion, grip strength, falls and adverse events, and hospital admissions compared to usual practice?	Improved disease and treatment burden, quality of life, cognition, fatigue, nutritional status, physical function capacity and ability, pain, sleep, patient enablement, medication self-efficacy and lower/fewer falls, healthcare utilization, and adverse events will be reported in the intervention arm compared to the control arm at 6-months	Disease burden	*T*_0_, *T*_6_	Linear regression
		Treatment burden	*T*_0_, *T*_6_	Linear regression
		Quality of life	*T*_0_, *T*_6_	Linear regression for all continuous outcomes, Logistic regression for Categorical outcomes and Count data models (e.g., Poisson/negative binomial regression, zero-inflated models) for count outcomes
			*T*_0_, *T*_6_	
		Cognition	*T*_0_, *T*_6_	
		Fatigue	*T*_0_, *T*_6_	
		Nutritional status	*T*_0_, *T*_6_	
		Physical function capacity and ability	*T*_0_, *T*_6_	
			*T*_0_, *T*_6_	
			*T*_0_, *T*_6_	
			*T*_0_, *T*_6_	
		Number of Falls	*T*_0_, *T*_6_	
		Pain	*T*_0_, *T*_6_	
		Sleep	*T*_0_, *T*_6_	
		Patient enablement	*T*_0_, *T*_6_	
		Medication self-efficacy	*T*_0_, *T*_6_	
		Healthcare resource utilization (number of hospitalizations, emergency department visits, primary care visits, proportion of patients with at least one hospitalizations)	*T*_0_, *T*_6_	
			*T*_0_, *T*_6_	
			*T*_0_, *T*_6_	
			*T*_0_, *T*_6_	
		Number of Serious adverse events and beneficial withdrawal	*T*_1_, *T*_3_, *T*_6_	
What is the cost-effectiveness of the structured medication discontinuation clinical pathway designed to reduce polypharmacy?	Not applicable	Cost per QALY	*T*_6_	Cost utility analysis
What is the experience of patients as they go through a structured medication discontinuation clinical pathway designed to reduce polypharmacy?	Not applicable	Lived experience with deprescribing process	*T*_6_	Descriptive analysis of Semi-structured interview/patient diaries
		Satisfaction with the intervention (5-point Likert scale)	*T*_6_	Descriptive statistics (M, SD)
		Satisfaction with care around medications (5-point Likert scale)	*T*_0_, *T*_6_	Linear regression
		Strengths and weaknesses of intervention	*T*_6_	Descriptive analysis
What are the experiences of the pharmacist and family physician of managing patients through the deprescribing process?	Not applicable	Lived experience with deprescribing process	*T*_6_	Thematic analysis of semi-structured interviews/field notes
		Confidence in medication discontinuation (5-point Likert scale)	*T*_0_, *T*_6_	Linear regression
		Five best/worst aspects of intervention	*T*_6_	Descriptive analysis
		Implementation processes	Early/late implementation	Descriptive analysis

*T*_*0*_ baseline, *T*_*3*_ month 3, *T*_*6*_ month 6

### Sample size

Sample size calculations for the RCT were performed by the team biostatistician (LT). We used data from our feasibility study to estimate sample size: mean number of medications of 7.7, a post-intervention reduction in mean (standard deviation) of 4.2 (± 2.5). There was consensus from the expert international polypharmacy advisory group that a mean reduction of 2 medications is clinically important [42].

A sample size of at least 160 per group is sufficient to detect a difference in mean number of medications of 2, assuming a starting mean of 7 and a static control group (power set at 80%, significance level 5%). It will also allow detection of an absolute difference in the proportion of patients with drug-related side effects of 15% (10% versus 25%) and provides adequate power for subgroup analysis for the primary outcome if proportions are 40% or more. Allowance has been made for 10% attrition; therefore, the final minimum recruitment target is 180 per group (total *N* = 360).

### Quantitative data analysis

The analysis and reporting of the results will follow the CONSORT guideline [71]. The process of patient selection and flow throughout the study will be summarized using a flow diagram. The analysis results of patient characteristics and baseline outcome variables (both primary and secondary) will be summarized using descriptive summary measures: expressed as mean (standard deviation) or median (minimum, maximum) for continuous variables depending on the distribution, and the number (percent) for categorical variables [72]. We will adopt an intention-to-treat (ITT) principle as our primary approach to analyze all outcomes and the people doing the analysis will be blind to study group allocation. Assuming data will be missing at random (MAR), we will also use multiple imputation [MI] through chained equations to impute missing data [73]. In total, five datasets will be imputed and pooled effect estimates (odds ratios for categorical outcomes, mean difference for continuous outcomes, and rate ratio for count outcomes) along with 95% confidence intervals and the associated *p* values will be reported. All statistical tests will be two-sided at the 0.05 level of significance. *P* values will be reported to three decimal places with values less than 0.001, reported as < 0.001. We will not adjust this for multiple analyses for secondary outcomes as we are considering them simply as exploratory. The patient will be the unit of analysis. Adopting an ITT principle, we will use linear regression to analyze both primary and secondary continuous outcomes. Analysis will be blinded. For categorical outcomes, we will use logistic regression. Count data models (e.g., Poisson/negative binomial regression, zero-inflated models) will be used to analyze the count outcomes. We will perform pre-specified subgroup analyses by adding an interaction term between intervention groups and the following subgroups: (1) age (≥ 85 versus < 85 years); and (2) frailty (frail versus severely frail, based on Rolfson et al., 2006 [60]). The interaction tests will be completely exploratory in nature and not adjusted for multiple testing. We examined articles in our review of reviews [74] of interventions to address polypharmacy in older adults living with multimorbidity that may have contained sub-analyses for age and frailty. We found one systematic review that contained a sub-analysis for mortality associated with deprescribing intervention subgroup analyses based on age [75]; and concluded there was no effect. Thus, we hypothesize that there will be no significant interaction effects for either sub-analysis. The interaction tests will be exploratory in nature and not adjusted for multiple testing. We will run sensitivity analyses to assess the robustness of the primary results, adopting a per-protocol approach. All analyses will be performed using SAS software 9.4 (Cary, NC).

We aim to book the family physician’s appointment within 1 week with a maximum of 2 weeks from the pharmacist appointment but acknowledge this may be difficult due to busy physician schedules or patient commitment changes. We aim to perform another sensitivity analysis to account for these differences in the timeline that may occur through linear regression.

### Qualitative data analysis

As some decisions are often emergent in qualitative research, we provide only brief details of our analytic approach to the qualitative piece to the study. Pseudonyms will be assigned to participants. Audiotapes for interviews will be transcribed verbatim. The transcripts will be coded inductively and independently by two research team members in three stages: open, axial, and selective [76]. The interview data generated (with patients, pharmacists, and family physicians), family physician and pharmacist field notes, and patient diaries will be analyzed using thematic analysis to identify, analyze, and report patterns in the data. Dedoose, an online data analysis application for qualitative and mixed methods research [77], will be used to support analysis. Consistent with recommendations, two strategies will be used to foster trustworthiness [78, 79]. First, two coders will independently code 100% of the data and meet to discuss initial patterns and themes and explore disagreement. Second, authenticity will be maintained by displaying direct quotations within the results.

### Adherence and fidelity issues

Adherence will not affect the ability to measure the main study outcomes in the way it does in other trials as it is part of the outcome measure itself. The need to restart medications is not seen as a protocol violation or bias, nor is it seen as a failure of treatment, rather it is seen as effective monitoring during the “pause and monitor” phase. Fidelity to the intervention will however be assessed using a checklist of critical elements (see Additional File 6).

### Contamination

Access to TaperMD is by individual record unique ID. Pharmacists and physicians will only be able to access TaperMD intervention patients. Access will only be available for control patients after they have passed through the 6-month data collection point.

### Loss to follow-up

We conservatively anticipate the loss to follow-up rate to be a maximum of 10%. Our experience in a discontinuation RCT of antidepressants in primary care showed only a 4% loss to follow-up over 18 months [80], and our feasibility studies in the same age group showed a loss to follow-up of less than 8%. Our TAPER feasibility RCT showed loss to follow-up of only 3 patients. We found regular contact, multiple alternate contact details, and a measured informed consent process, particularly ensuring participants are clearly able to be available for follow-up, are helpful in minimizing such losses. In our feasibility study, we found it unhelpful to recruit participants during the “snowbird” period in winter who were traveling outside of Canada for an extended time as many older Canadians do. Perceived insurance restrictions mean they are not willing to change their medication in the period prior to leaving. The participants lost to follow-up will be classified as lost to follow-up due to an inability to contact, elected to no longer be part of any further follow-up (withdrawal of consent), or unable to participate because of ill health. In cases where participants who are lost to follow-up do not explicitly withdraw consent, medication and health care utilization data will still be collected from patient charts via chart audit where possible.

### Safety and monitoring

Details of any clinically adverse symptoms or effects reported to researchers at research data collection points will be securely provided to their family physician and pharmacist at that time, either by personal communication from the research team or electronically to a secure clinic fax number as appropriate. These processes did not compromise the blinding in our feasibility study [42]. Any such adverse effects will be addressed within the usual primary care clinical setting by their family physician who will be responsible for the clinical follow-up medication pause or dose reduction. Clinical follow-up will occur as determined by the monitoring requirements for the particular drugs discontinued.

If the patient experiences a serious adverse event (SAE), defined in a previous section on adverse events, a local SAE record will be completed and reported to the family physician within 24 h if they are unaware. The study team will notify the research ethics board of any SAE thought to be related to the study, using the standard SAE form. The form will be signed off by the principal investigator within 48 h. No formal auditing of trial conduct will take place. There will not be a data monitoring committee involved with the trial as there are no stopping rules for the trial and no planned interim analyses. Research staff will be responsible for overseeing study progress. Regular contact among study team members and clinic staff will be made to discuss and resolve any issues thought to be related to study conduct, and if any protocol or process changes are required these will be recorded as previously stated. There are no provisions for post-trial or ancillary care, as patients will continue to receive care as necessary from their physician throughout and following participation the trial. Furthermore, the study is low-risk and no harms from trial participation are anticipated.

### Dissemination policy

All results of the trial will be published, and there are no publication restrictions.

## Discussion

This study protocol describes an RCT designed to assess the effect of TAPER in reducing polypharmacy. Polypharmacy is associated with negative health outcomes. It is not known whether these effects are reversible if polypharmacy is reduced. The study is designed to assess the effect of TAPER on reducing polypharmacy and the extent to which this affects selected health outcomes, based on the negative associations with polypharmacy found in non-randomized studies. This study will be a large multi-centered single-blinded RCT.

Our study so far is unique in its design in aiming to introduce an operationalized structured clinical pathway aimed to tackle polypharmacy in a primary care setting while at the same time recording patient’s goals and priorities for treatment. A variety of explicit tools and deprescribing guidelines are available to flag medications that may be inappropriate or suitable for discontinuation in older adults. In addition, there are several studies evaluating pharmacist and physician collaboration to improve medication management in primary care [18]. However, very few pathways integrate all of these in one place and across all medications simultaneously, and fewer operationalize patients’ views and consideration of patients’ actual goals and priorities for treatment.

A strength of this trial design is its pragmatic nature and the setting where the evidence will be applied. The pathway and tool are immediately scalable and the results of the trial, if successful, will be directly translatable into clinical practice. Evidence from this study will help understand the provision of a systematic approach to considering reduction of the burden of polypharmacy, as well as providing a systematic clinical pathway for implementation including integration of available evidence tools, active monitoring, and reinstatement criteria.

While there are many studies associating negative health outcomes with polypharmacy, these result in observational data only and do not prove that outcomes improve when polypharmacy is reduced. If successful, our aim in testing this intervention is to lay the foundation for this model to be incorporated and become a part of routine preventive care in older adults.

## Trial status

Recruitment for the trial started in June, 2018, and is expected to continue recruitment until late 2021. This is protocol version 1, July 31, 2020.

## Supplementary Information

**Additional file 1.** PDF. Spirit 2013 Checklist.

**Additional file 2.** PDF. TIDier Checklist.

**Additional file 3.** PDF. TaperMD. A list of the questions on the TaperMD web-based platform.

**Additional file 4.** PDF. Quantitative patient outcomes measure descriptions. A list of the primary and secondary outcomes, descriptions of the measures and the psychometric properties of the measures.

**Additional file 5.** PDF. Data collection schedule. A diagram outlining the proposed timeline of the study.

**Additional file 6.** PDF. Site fidelity to TAPER-RCT model. The checklist that will be used to measure a site’s adherence to the TAPER-RCT protocol.

## Data Availability

The study team will have full access to the dataset. Data will be made available once planned analyses and publications by the study team are completed, as well as online training materials. Anonymized patient-level data will be made available for meta-analyses on request. There are no plans to grant access to statistical code.

## Abbreviations

API: Application programming interface
CTCAE: Common Terminology Criteria for Adverse Events
EMR: Electronic medical records
ITT: Intention-to-treat
LOCF: Last-Observation-Carried-Forward
MAR: Missing at random
MI: Multiple imputation
MMSE: Mini-Mental Status Examination
MNASF: Mini-Nutritional Assessment Short-Form
PEI: Patient Enablement Instrument
PIM: Potentially inappropriate medication
RCT: Randomized controlled trial
REDCap: Research Electronic Data Capture
SAE: Serious adverse event
SEAMS: Self-Efficacy for Appropriate Medication Use scale
STOPP: Screening Tool of Older Person’s Prescriptions
TAPER: Team Approach to Polypharmacy Evaluation and Reduction
QALY: Quality-adjusted life years
