# Supplementary material for: Team approach to polypharmacy evaluation and reduction: study protocol for a randomized controlled trial
Source: Trials. 2021 Oct 26;22:746. doi: 10.1186/s13063-021-05685-9 (PMC8549321; doi:10.1186/s13063-021-05685-9)
Supplement: Supplementary file 2 — Additional file 2. PDF. TIDier Checklist. [file 13063_2021_5685_MOESM2_ESM.pdf]

Additional file 2: TIDier (Template for Intervention Description and Replication) Checklist

Item Number	Item	Where located	
		Primary paper (page or appendix)	Other† (details)
**Brief Name**			
1	Provide the name of a phrase that describes the intervention.	6	
**Why**			
2	Describe any rationale, theory, or goal of the elements essential to the intervention.	7-8	
**What**			
3	Materials: Describe any physical or informational materials used in the intervention, including those provided to participants or used in intervention delivery or in training of intervention providers. Provide information on where the materials can be accessed (e.g. online appendix, URL).	12-15, 30, Additional file 3, Additional file 6	
4	Procedures: Describe each of the procedures, activities, and/or processes used in the intervention, including any enabling or support activities.	11-17	
**Who Provided**			
5	For each category of intervention provider (e.g., psychologist, nursing assistant), describe their expertise, background and any specific training given.	11-12	
**How**			
6	Describe the modes of delivery (e.g., face-to-face or by some other mechanism, such as internet or telephone) of the intervention and whether it was provided individually or in a group.	14-16	
Where			
7	Describe the type(s) of location(s) where the intervention occurred, including any necessary infrastructure or relevant features.	9	
**When and How Much**			

8	Describe the number of times the intervention was delivered and over what period of time including the number of sessions, their schedule, and their duration, intensity or dose.	13-16, Additional file 5	
**Tailoring**			
9	If the intervention was planned to be personalized, titrated or adapted, then describe what, why, when and how.	14-16	TAPER plan is tailored to patient
**Modifications**			
10ǂ	If the intervention was modified during the course of the study, describe the changes (what, why, when and how).	Not applicable	Protocol
**How Well**			
11	Planned: If intervention adherence or fidelity was assessed, describe how and by whom, and if any strategies were used to maintain or improve fidelity, describe them.	12, 25, Additional file 6	
12ǂ	Actual: If intervention adherence or fidelity was assessed, describe the extent to which the intervention was delivered as planned.	Not applicable	Protocol

N/A = an item is not applicable for the intervention being described

ǂ If completing the TIDieR checklist for a protocol, these items are not relevant to the protocol and cannot be described until the study is complete.
