# Supplementary material for: Team approach to polypharmacy evaluation and reduction: study protocol for a randomized controlled trial
Source: Trials. 2021 Oct 26;22:746. doi: 10.1186/s13063-021-05685-9 (PMC8549321; doi:10.1186/s13063-021-05685-9)
Supplement: Supplementary file 4 — Additional file 4. PDF. Quantitative patient outcomes measure descriptions. A list of the primary and secondary outcomes, descriptions of the measures and the psychometric properties of the measures. [file 13063_2021_5685_MOESM4_ESM.pdf]

Additional file 4: Quantitative patient outcome measure descriptions

Primary outcome: number of medications

**Included medications**	**Excluded medications**
Long-term prescribed medications	Locally-acting topicals
Systemically-acting topicals	Eye drops
Puffers	Nasal sprays
Regular injections	Non-prescription cannabis
Long-term nicotine replacement therapy	
Each active ingredient of a combination drug	
Over the counter drugs and supplements are included if it was purchased on recommendation by or prescribed by a health-care practitioner (e.g., low dose ASA).	

Secondary outcomes

**Construct**	**Measure**	**Measure description**	**Validity and reliability information**
Disease burden	Disease Burden survey [1]	From a list of common chronic conditions, respondents are asked to indicate which conditions they have, and for those conditions present, the degree to which the condition affects their daily activity from “not at all” to “a lot”.	Negative relationships have been shown between this scale and health status and physical functioning, providing some evidence of validity of the scale [1].
Treatment burden	Brief Treatment Burden Scale [2]	Respondents will be asked to rate their level of difficulty they have with ten different treatment-related tasks (i.e., taking lots of medications, seeing lots of	This scale has demonstrated good validity, reliability, and sensitivity to change for patients with multimorbidity.

Construct	Measure	Measure description	Validity and reliability information
		different health professionals, having to rely on help from family and friends). The scale used to rate their difficulty is from “not difficult” to “extremely difficult”.	
Quality of life	EuroQol five dimension (EQ5D-5L) [3]	Respondents rate 5 domains of health (mobility, self-care, usual activities, pain/discomfort and finally anxiety/depression) on a 5-point scale. Scores range from 0 to 1, with higher scores representing higher quality of life [3, 4]. In addition, a single-item assesses respondents’ overall level of health state from 0-100 (‘worst imaginable health’ to ‘best imaginable health’).	EQ5D-5L is a widely used survey, which has demonstrated validity and reliability [5, 6].<br><br>It is suitable for cost utility analysis and will be used in our economic analysis [3].
	Short Form Health Survey (SF36v2) [7]	Eight domains, including role limitations due to physical health problems, bodily pain, general health, vitality, social functioning, role limitations due to emotional problems, physical functioning and mental health are rated. Scores range from a scale of 0-100, with higher values representing higher quality of life for the domain.	The SF-36 is considered a valid, reliable, and concise generic measure of health [8].
Cognition	Mini Mental Status Examination [9]	The examination consists of 11 items that aim to measure five areas of cognitive functions: orientation, registration, attention, calculation, recall and language. Scores of each item summed, scores range	It has also been used to show change in studies of discontinuation of antipsychotic medication in older adults [10, 11].

Construct	Measure	Measure description	Validity and reliability information
		from 0 to 30 [9]. Scores of 0-9, 10-19, 20-25, 26-30 represent different degrees of impairment, including severe, moderate, mild, and potentially normal, respectively	
Fatigue	Avlund mobility-tiredness subscale [12]	This is a 6-item subscale that asks participants about fatigue with performing six activities [12]. The scoring system is based on a yes (1) and no (0) answers, which are added for a sum (0-6 points). Higher values indicate a greater degree of fatigue.	The scale has been validated and used with younger-older populations and has shown good reliability, validity, and predictive value [13, 14].
Nutritional status	Mini Nutritional Assessment Short-Form [15, 16]	The survey has 6 items, with each having a score that are summed together. The scores range from 0-14, with higher scores indicating a better nutritional status.	The scale has been shown to be a well validated tool for nutritional assessment in older populations and is suitable for use in ambulatory populations [15, 16] and frail older adults [16].
Physical function capacity and ability	Mänty structured interview [17]	Responses define preclinical, minor or major mobility limitation as well as no mobility limitation for three common tasks (walking 2 km, walking 0.5 km and climbing 1 flight of stairs).	The survey is a valid measure to capture the early signs of disability and identify those at high risk for future disability [17].
	Timed up and go [18]	Participants are instructed to sit in a chair, and on the word ‘go’, stand up from the chair and walk at a safe and comfortable pace to a mark on the floor (3m away), turn around, then return to the chair and sit down	This test is a valid and reliable measure of lower body strength and function as well as balance, with normative reference values stratified by age [18, 19].

Construct	Measure	Measure description	Validity and reliability information
	Grip strength [20]	Grip strength will be measured using a JAMAR hand dynamometer. A protocol involving forearm supported grip strength based on [21], but alternating between right and left hands to prevent fatigue will be used as a measure of grip strength. The mean of three trials of each hand will be used in data analyses.	Grip strength measures have excellent test-retest reliability and normative distributions have been developed, stratified by age and sex [20-22].
	Global rating of change for balance [23]	Participants are asked to assess their current balance ability compared to their status at a previous time point (6-months previous), and then indicate the difference on a visual analogue scale from -5 ('very much worse') to 5 ('very much better'), with a midpoint of 0 ('unchanged').	Global ratings of change have been used to measure patient-detected change on a wide variety of constructs, showing high test-retest reliability and face-validity [23].
Falls	Not applicable	Number of significant falls (defined as falls resulting in medical consultation or treatment) will be self-reported.	Not applicable
Pain	Brief Pain Inventory [24]	Only those items contributing to the scores of the two subscales (pain interference and pain severity) will be used.	The Brief Pain Inventory has shown convergent validity and discriminative validity [25].
Sleep	Sleep item from the 15-D scale [26]	1 question rating sleep quality from "ability to sleep normally" to "severe problems with sleeping"	The 15-D has been shown to be valid in a variety of population [26] including, chronic pain [27] and Parkinson's disease [28].
Patient enablement	A modified version of the validated Patient Enablement Instrument	Consists of 6 questions that revolve around patient understanding of, and coping with, health issues as a result of a consultation	The Patient Enablement Instrument has presented satisfactory measurement properties including

Construct	Measure	Measure description	Validity and reliability information
	developed in primary care in the United Kingdom [29]. The stem "after a usual visit with your family doctor, do you feel that you are…" was used instead of "as a result of your visit to the doctor today…"	with a healthcare provider. The total score would range from 0-12, with a higher score indicating a stronger patient enablement.	cross-cultural validity, reproducibility, and internal consistency [30].
Medication self-efficacy	Self-Efficacy for Appropriate Medication Use scale [31]	Thirteen items are rated as "not confident", "somewhat confident" or "very confident". Means for two subscales, self-efficacy for taking medications under difficult circumstances and self-efficacy for continuing to take medications when circumstances surrounding medication-taking are uncertain, will be calculated.	The measure has shown to be a reliable and valid instrument to measure self-efficacy in medication management and our own systematic review suggests is the highest quality measure of medication self-efficacy available at this time [32].
Healthcare utilization	Not applicable	Hospital admissions, and emergency department/urgent care and primary care visits will be collected from patient electronic medical records or other administrative data	Not applicable

References

1. Bayliss EA, Ellis JL, Steiner JF. Seniors' self-reported multimorbidity captured biopsychosocial factors not incorporated into two other data-based morbidity measures. J Clin Epidemiol. 2009;62(5):550-7 e1.
2. Duncan P, Murphy M, Man MS, Chaplin K, Gaunt D, Salisbury C. Development and validation of the Multimorbidity Treatment Burden Questionnaire (MTBQ). BMJ Open. 2018;8(4):e019413.
3. Rabin R, de Charro F. EQ-5D: a measure of health status from the EuroQol Group. Ann Med. 2001;33(5):337-43.

4. EuroQol Group. Measuring Self-Reported Population Health: An International Prespective based on EQ-5D: Springer; 2004.
5. Hurst NP, Kind P, Ruta D, Hunter M, Stubbings A. Measuring health-related quality of life in rheumatoid arthritis: validity, responsiveness and reliability of EuroQol (EQ-5D). Br J Rheumatol. 1997;36(5):551-9.
6. Nolan CM, Longworth L, Lord J, Canavan JL, Jones SE, Kon SS, et al. The EQ-5D-5L health status questionnaire in COPD: validity, responsiveness and minimum important difference. Thorax. 2016;71(6):493-500.
7. RAND Corporation. 36-Item Short Form Survey (SF-36). 2021. https://www.rand.org/health-care/surveys_tools/mos/36-item-short-form.html. Accessed 23 March 2021.
8. Failde I, Ramos I. Validity and reliability of the SF-36 Health Survey Questionnaire in patients with coronary artery disease. J Clin Epidemiol. 2000;53(4):359-65.
9. Folstein MF, Folstein SE, McHugh PR. "Mini-mental state". A practical method for grading the cognitive state of patients for the clinician. J Psychiatr Res. 1975;12(3):189-98.
10. Jyrkka J, Enlund H, Lavikainen P, Sulkava R, Hartikainen S. Association of polypharmacy with nutritional status, functional ability and cognitive capacity over a three-year period in an elderly population. Pharmacoepidemiol Drug Saf. 2011;20(5):514-22.
11. Ballard C, Orrell M, YongZhong S, Moniz-Cook E, Stafford J, Whittaker R, et al. Impact of Antipsychotic Review and Nonpharmacological Intervention on Antipsychotic Use, Neuropsychiatric Symptoms, and Mortality in People With Dementia Living in Nursing Homes: A Factorial Cluster-Randomized Controlled Trial by the Well-Being and Health for People With Dementia (WHELD) Program. Am J Psychiatry. 2016;173(3):252-62.
12. Avlund K, Kreiner S, Schultz-Larsen K. Functional ability scales for the elderly: A validation study. Eur J Public Health. 1996;6(1):35-42.
13. Brühl A, Avlund K. Validity and internal consistency of mobility scales for healthy older people in Germany. J Clin Gerontology Geriatrics. 2012;3(1):29-35.
14. Manty M, Ekmann A, Thinggaard M, Christensen K, Avlund K. Indoor mobility-related fatigue and muscle strength in nonagenarians: a prospective longitudinal study. Aging Clin Exp Res. 2014;26(1):39-46.
15. Rubenstein LZ, Harker JO, Salva A, Guigoz Y, Vellas B. Screening for undernutrition in geriatric practice: developing the short-form mini-nutritional assessment (MNA-SF). J Gerontol A Biol Sci Med Sci. 2001;56(6):M366-72.
16. Kaiser MJ, Bauer JM, Ramsch C, Uter W, Guigoz Y, Cederholm T, et al. Validation of the Mini Nutritional Assessment short-form (MNA-SF): a practical tool for identification of nutritional status. J Nutr Health Aging. 2009;13(9):782-8.
17. Manty M, Heinonen A, Leinonen R, Tormakangas T, Sakari-Rantala R, Hirvensalo M, et al. Construct and predictive validity of a self-reported measure of preclinical mobility limitation. Arch Phys Med Rehabil. 2007;88(9):1108-13.
18. Podsiadlo D, Richardson S. The timed "Up & Go": a test of basic functional mobility for frail elderly persons. J Am Geriatr Soc. 1991;39(2):142-8.
19. Bohannon RW. Reference values for the timed up and go test: a descriptive meta-analysis. J Geriatr Phys Ther. 2006;29(2):64-8.
20. Bohannon RW. Test-Retest Reliability of Measurements of Hand-Grip Strength Obtained by Dynamometry from Older Adults: A Systematic Review of Research in the PubMed Database. J Frailty Aging. 2017;6(2):83-7.
21. Mathiowetz V, Weber K, Volland G, Kashman N. Reliability and validity of grip and pinch strength evaluations. J Hand Surg Am. 1984;9(2):222-6.

22. Desrosiers J, Bravo G, Hebert R, Dutil E. Normative data for grip strength of elderly men and women. Am J Occup Ther. 1995;49(7):637-44.
23. Kamper SJ, Maher CG, Mackay G. Global rating of change scales: a review of strengths and weaknesses and considerations for design. J Man Manip Ther. 2009;17(3):163-70.
24. Cleeland CS, Ryan KM. Pain assessment: global use of the Brief Pain Inventory. Ann Acad Med Singap. 1994;23(2):129-38.
25. Song CY, Lin SF, Huang CY, Wu HC, Chen CH, Hsieh CL. Validation of the Brief Pain Inventory in Patients With Low Back Pain. Spine (Phila Pa 1976). 2016;41(15):E937-E42.
26. Sintonen H. The 15D instrument of health-related quality of life: properties and applications. Ann Med. 2001;33(5):328-36.
27. Vartiainen P, Mantyselka P, Heiskanen T, Hagelberg N, Mustola S, Forssell H, et al. Validation of EQ-5D and 15D in the assessment of health-related quality of life in chronic pain. Pain. 2017;158(8):1577-85.
28. Haapaniemi TH, Sotaniemi KA, Sintonen H, Taimela E. The generic 15D instrument is valid and feasible for measuring health related quality of life in Parkinson's disease. J Neurol Neurosurg Psychiatry. 2004;75(7):976-83.
29. Howie JG, Heaney DJ, Maxwell M, Walker JJ. A comparison of a Patient Enablement Instrument (PEI) against two established satisfaction scales as an outcome measure of primary care consultations. Fam Pract. 1998;15(2):165-71.
30. Remelhe M, Teixeira PM, Lopes I, Silva L, Correia de Sousa J. The modified patient enablement instrument: a Portuguese cross-cultural adaptation, validity and reliability study. NPJ Prim Care Respir Med. 2017;27:16087.
31. Risser J, Jacobson TA, Kripalani S. Development and psychometric evaluation of the Self-efficacy for Appropriate Medication Use Scale (SEAMS) in low-literacy patients with chronic disease. J Nurs Meas. 2007;15(3):203-19.
32. Lamarche L, Tejpal A, Mangin D. Self-efficacy for medication management: a systematic review of instruments. Patient Prefer Adherence. 2018;12:1279-87.
