# Supplementary material for: Team approach to polypharmacy evaluation and reduction: study protocol for a randomized controlled trial
Source: Trials. 2021 Oct 26;22:746. doi: 10.1186/s13063-021-05685-9 (PMC8549321; doi:10.1186/s13063-021-05685-9)
Supplement: Supplementary file 5 — Additional file 5. PDF. Data collection schedule. A diagram outlining the proposed timeline of the study. [file 13063_2021_5685_MOESM5_ESM.pdf]

Additional file 5: Data collection schedule

Implementation sites trained	A	
Recruitment of participants	B	
Baseline data collection on pharmacists, family physicians	C	
Randomization of participants	D	
	Intervention	Control
Baseline data collection on patients	E	E
Intervention	F	
Monitoring	G	
Diaries, semi-structured interviews with sub-set of patients	H	
1-week follow-up	I	I
3-month follow-up	J	J
6-month follow-up of research outcomes	K	K
Follow-up data collection on pharmacists, family physicians	L	L

A	Main site: March 2018-June 2018 ; Implementation site(s): October 2019-January 2020
B	Rolling recruitment, June 2018-June 2020
C	Main site June 2019-July 2019 ; Implementation site(s) October 2019-January 2020
D	Randomization one eligibility confirmed and consent signed, rolling fashion, June 2018-June 2020
E	Baseline data collection, rolling fashion, 24 months, June 2018-June 2020
F	Intervention (pharmacist and physician appointments), rolling fashion, June 2018-December 2020
G	Patient monitoring following intervention, rolling fashion, June 2018-January 2021
H	Qualitative piece (diaries, semi-structured interviews), June 2018-June 2020
I	One-week follow-up, June 2018-July 2020
J	3-month follow-up, September 2018-September 2020
K	Six-month data collection, rolling fashion, 24 months, December 2018-December 2020
L	Delayed (wait-list) intervention for control patients, rolling fashion, December 2018-December 2020
