# Supplementary material for: Team approach to polypharmacy evaluation and reduction: study protocol for a randomized controlled trial
Source: Trials. 2021 Oct 26;22:746. doi: 10.1186/s13063-021-05685-9 (PMC8549321; doi:10.1186/s13063-021-05685-9)
Supplement: Supplementary file 6 — Additional file 6. PDF. Site fidelity to TAPER-RCT model. The checklist that will be used to measure a site’s adherence to the TAPER-RCT protocol. [file 13063_2021_5685_MOESM6_ESM.pdf]

Additional File 6: Site Fidelity to TAPER-RCT Model

Program components	Program outcomes and outputs	Indicator	Data Source	Analysis	Evaluation
Team Approach [1, 2]	Team-based deprescribing intervention	The team includes a family physician, pharmacist, and patient (or their family member)	Discussion with practice clinic team	Checklist: Yes/No	Yes = 1<br>No = 0
Pharmacist appointment	Appointment with a pharmacist	Patient is scheduled for an appointment with the pharmacist	EMR schedule or discussion with pharmacist regarding scheduling	Checklist: Yes/No	Yes = 1<br>No = 0
	Medication reconciliation is performed	An in-depth medication reconciliation is performed	TaperMD	Checklist: Yes/No	Yes = 1<br>No = 0
	Plan is initiated	The pharmacist suggests a plan of action and discusses the plan with patient until a decision to proceed is made	TaperMD	Checklist: Yes/No	Yes = 1<br>No = 0
Use of TaperMD	Baseline medications are entered into TaperMD	Baseline medications are entered into TaperMD	TaperMD	Checklist: Yes/No	Yes = 1<br>No = 0
	Goals and symptom priorities entered into TaperMD	Goals and symptom priorities are entered into TaperMD	TaperMD	Checklist: Yes/No	Yes = 1<br>No = 0
	Machine screen performed during the pharmacist appointment	Medications are “machine screened”	TaperMD	Checklist: Yes/No	Yes = 1<br>No = 0
	A plan of action is developed	A plan of action is created, which may or may not include “pause and monitor” of a medication	TaperMD	Checklist: Yes/No	Yes = 1<br>No = 0
Physician appointment	Appointment with physician	Patient is scheduled for an appointment with their family physician	EMR	Checklist: Yes/No	Yes = 1<br>No = 0

Program components	Program outcomes and outputs	Indicator	Data Source	Analysis	Evaluation
	Action plans are finalized	The family physician finalizes (either verifying or modifying) the plan of action proposed by the pharmacist (with patient's input)	TaperMD, EMR	Checklist: Yes/No	Yes = 1<br>No = 0
Ongoing patient monitoring	Monitoring plan is created if appropriate	A monitoring plan is created with patients deemed to require monitoring	TaperMD	Checklist: Yes/No	Yes (or not applicable) = 1<br>No = 0
	Patients monitored according to plan	Monitoring plan implemented	TaperMD, EMR	Checklist: Yes/No	Yes (or need not applicable) = 1<br>No = 0<br>Need not applicable
	Plan adjusted as necessary	The action plan is adjusted as necessary depending on the results from patient monitoring	TaperMD, EMR	Checklist: Yes (or adjustment not needed)/No	Yes (or not applicable) = 1<br>No = 0
	**TOTAL**				**/13**

References

1. Davies SM, Geppert J, McClellan M, McDonald KM, Romano PS, Shojania KG. Refinement of the HCUP Quality Indicators: Agency for Healthcare Research and Quality (US), Rockville (MD); 2001.
2. Dillman DA. Mail and web-based survey: The tailored design method. NY: John Wiley & Sons. 2000.
